# Rationally Designed TLR4 Ligands for Vaccine Adjuvant Discovery

**DOI:** 10.1128/mBio.00492-17

**Published:** 2017-05-09

**Authors:** Kelsey A. Gregg, Erin Harberts, Francesca M. Gardner, Mark R. Pelletier, Corinne Cayatte, Li Yu, Michael P. McCarthy, Jason D. Marshall, Robert K. Ernst

**Affiliations:** a Department of Microbial Pathogenesis, University of Maryland School of Dentistry, Baltimore, Maryland, USA; b Statistical Sciences, MedImmune, Gaithersburg, Maryland, USA; c Vaccine Platform Group, MedImmune, Gaithersburg, Maryland, USA; University of Illinois at Chicago

**Keywords:** TLR4, adjuvants, immunomodulation, innate immunity, lipid A, lipopolysaccharide, memetic, Toll-like receptors

## Abstract

Adjuvant properties of bacterial cell wall components like MPLA (monophosphoryl lipid A) are well described and have gained FDA approval for use in vaccines such as Cervarix. MPLA is the product of chemically modified lipooligosaccharide (LOS), altered to diminish toxic proinflammatory effects while retaining adequate immunogenicity. Despite the virtually unlimited number of potential sources among bacterial strains, the number of useable compounds within this promising class of adjuvants are few. We have developed bacterial enzymatic combinatorial chemistry (BECC) as a method to generate rationally designed, functionally diverse lipid A. BECC removes endogenous or introduces exogenous lipid A-modifying enzymes to bacteria, effectively reprogramming the lipid A biosynthetic pathway. In this study, BECC is applied within an avirulent strain of *Yersinia pestis* to develop structurally distinct LOS molecules that elicit differential Toll-like receptor 4 (TLR4) activation. Using reporter cell lines that measure NF-κB activation, BECC-derived molecules were screened for the ability to induce a lower proinflammatory response than *Escherichia coli* LOS. Their structures exhibit varied, dose-dependent, TLR4-driven NF-κB activation with both human and mouse TLR4 complexes. Additional cytokine secretion screening identified molecules that induce levels of tumor necrosis factor alpha (TNF-α) and interleukin-8 (IL-8) comparable to the levels induced by phosphorylated hexa-acyl disaccharide (PHAD). The lead candidates demonstrated potent immunostimulation in mouse splenocytes, human primary blood mononuclear cells (PBMCs), and human monocyte-derived dendritic cells (DCs). This newly described system allows directed programming of lipid A synthesis and has the potential to generate a diverse array of TLR4 agonist candidates.

## IMPORTANCE

There is an urgent need to develop effective vaccines against infectious diseases that continue to be major causes of morbidity and mortality worldwide. Making effective vaccines requires selecting an adjuvant to strengthen an appropriate and protective immune response. This work describes a practical method, bacterial enzymatic combinatorial chemistry (BECC), for generating functionally diverse molecules for adjuvant use. These molecules were analyzed in cell culture for their ability to initiate immune stimulatory activity. Several of the assays described herein show promising *in vitro* cytokine production and costimulatory molecule expression results, suggesting that the BECC molecules may be useful in future vaccine preparations.

## INTRODUCTION

Contemporary prophylactic infectious disease vaccines often combine a well-characterized recombinant protein antigen with an adjuvant to increase the immunogenic response. To date, vaccine adjuvants have been developed using an empirical trial-and-error approach (1, 2). These efforts have identified adjuvants that compensate for poor antigen immunogenicity, increase vaccine stability, and reduce the amount of an antigen required for protection (3, 4). Previous adjuvants licensed in human vaccines, such as alum and oil-in-water emulsions, skew the resulting immune response toward T-helper 2 (Th2) immunity (5–7). Th2 responses are characterized by the production of interleukin-4 (IL-4) and antibody isotypes/subclasses that strongly promote complement fixation and opsonization of pathogens, an ideal response to combat extracellular pathogens. However, a Th2 response is not the most effective immune response against intracellular pathogens. A Th1 immune response, characterized in part by cytotoxic T-cell responses, can be more effective against intracellular pathogens. Current vaccine development strives to initiate and propagate an appropriate immune response for each target pathogen. To address these limitations, future vaccines may require improved adjuvants that stimulate defined innate immune pathways, including the Toll-like receptor (TLR) pathways (2). The canonical Toll-like receptor 4 (TLR4) signaling cascade is initiated when lipid A (the membrane anchor of lipopolysaccharide [LPS]) is bound by the extracellular region of CD14, which complexes with MD2 and binds to membrane-bound TLR4. Dimerization of these molecules with another lipid A-MD2-TLR4 complex creates a functional TLR4 signaling complex. Binding of a TLR4 agonist like lipid A initiates an innate immune response that can drive the development of antigen-specific acquired immunity.

Currently, the TLR4 agonists 3-*O*-deacyl-4′-monophosphoryl lipid A (MPLA) and aminoalkyl glucosaminide phosphates (AGPs) are well-studied examples of TLR4 ligand (TLR4L) adjuvants that can promote a Th1 (cellular)-biased immune response (8). These molecules show great promise for use as immunotherapeutic adjuvants to potentiate host responses in component vaccines. Recently, Fendrix, a vaccine for hepatitis B, and Cervarix, a vaccine for human papillomavirus, were licensed for use; both are adjuvanted with AS04, a combination of alum and MPLA (9–12). Additionally, phosphorylated hexa-acyl disaccharide (PHAD), a synthetic MPLA variant TLR4L, has advanced into the clinic for testing in several component vaccines (13–16). The production of MPLA requires extensive chemical modification of biologically derived LPS, which results in large batch-to-batch variability. While the synthetic production of PHAD is less variable than the production of MPLA, it is costly and not ideal for the large-scale production needed. The labor-intensive nature of *de novo* synthesization of AGPs is also not ideal and is prohibitive for their widespread use in vaccines. Alternative technologies that address these concerns of variability and production are needed for continuing vaccine development. The goals of next-generation vaccine adjuvant development are 2-fold: efficient manufacture and the ability to elicit a defined balance of antibody subtypes and cellular immune responses tailored for each vaccine (e.g., enhanced neutralizing antibodies or cytolytic T cells).

In this paper, we describe bacterial enzymatic combinatorial chemistry (BECC), a method to develop novel TLR4Ls by harnessing lipid A biosynthetic pathways from diverse bacteria. BECC removes or introduces lipid A-modifying enzymes into Gram-negative bacteria to alter the phosphate, acyl chain, and carbohydrate contents of lipid A. This includes the heterologous expression and homologous deletion of regulatory, acyltransferase, deacylase, phosphatase, and glycosyl transferase genes to create novel lipid A structures. BECC allows the creation of partial agonist TLR4Ls with potential for use as vaccine adjuvants; these TLR4Ls promote an effective adaptive immune response without the adverse inflammatory effects observed with potent TLR4 agonists like *Escherichia coli* lipid A. Here, we describe BECC as a mechanism to generate novel TLR4Ls and the employment of *in vitro* screening methods to identify molecules that have may potential for use in next-generation vaccines.

## RESULTS

### Synthesis of TLR4L structure.

**FIG 1:**
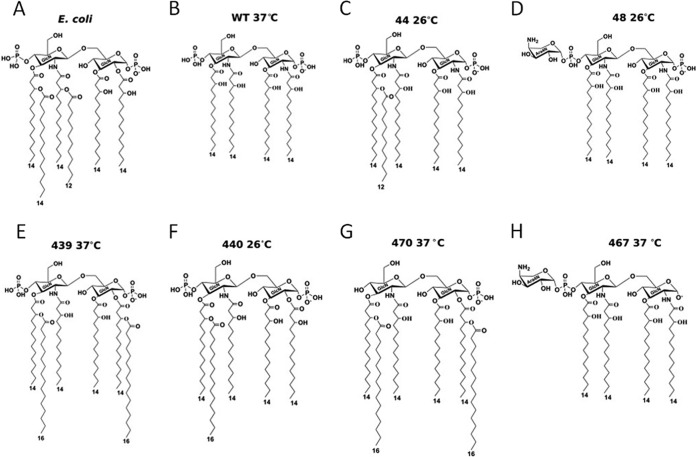
BECC-generated TLR4 ligands. Lipid A structures are shown for highly proinflammatory *E. coli* LOS (LOS_Ec_) (A), noninflammatory LOS of *Y. pestis* KIM6+ grown at 37°C (wild type [WT] LOSYp@37) (B), and BECC-generated LOS mutants LOSYp44@26 (C), LOSYp48@26 (D), LOSYp439@37 (E), LOSYp440@26 (F), LOSYp470@37 (G), and LOSYp467@37 (H). (C to H) The first number at the top of each panel refers to the strain/molecule number given during development (described in Table 1) and the temperature at which cultures were grown is indicated.

Next-generation vaccines require effective adjuvants with the potential to fine-tune the balance between activating an appropriate immune response and avoiding excessive adverse host reactions, such as inflammation. Lipid A is a complex, species-specific structure composed of carbohydrates, phosphates, and acyl groups (Fig. 1). Different lipid A configurations can alter TLR4 agonist activity (e.g., tetra-acylated lipid A molecules tend to be weaker agonists than hexa-acylated ones). Here, we show a mechanism for the synthesis and characterization of TLR4 mimetics using bacterial enzymatic combinatorial chemistry (BECC). BECC allows us to effectively reprogram the lipid A biosynthetic pathway and has the potential to achieve the delicate balance between activating immunity and avoiding excessive adverse host reactions.

In these studies, we engineered modified lipooligosaccharide (LOS) structures in an attenuated (biosafety level 2 [BSL2]) *Yersinia pestis* background to produce novel LOS/lipid A structures. When grown at a mammalian temperature, 37°C, *Y. pestis* lipid A is not proinflammatory in humans due to the bisphosphorylated, tetra-acylated lipid structure produced. This lipid A represents a structural and immunological blank slate for the addition of acyl chains and other modifications. When *Y. pestis* is grown at 26°C, the temperature of growth inside the flea vector, the lipid A synthesis is shifted to more proinflammatory penta- and hexa-acylated structures (Fig. 1) (17). Modification of these base *Y. pestis* lipid A structures using BECC and temperature control offers the potential to fine tune the balance between Th1 and Th2 immune responses, providing a robust mechanism for customizing vaccine adjuvant design.

Novel TLR4Ls were generated by BECC mutations in *Yersinia pestis* strain KIM6+ (pCD1^−^), an attenuated (BSL2) strain. We extracted, purified, and screened combinations of enzyme deletions and additions from a set of 6 lipid A-modifying mutations for differential TLR4 activation (Table 1; Fig. 1). BECC-derived strains were assigned unique identification numbers upon creation that are carried through in this paper. Also included in the name is the temperature at which the bacteria were grown: for example, 44@26 is strain 44 grown at 26°C. Included among the mutations selected was the deletion of *phoP*, encoding a protein in the PhoPQ two-component regulatory system that has been shown to affect genes responsible for the addition of terminal galactose to LOS and aminoarabinose to lipid A (18, 19). The acyl groups of lipid A are bound in a hydrophobic pocket of MD2, and an aminoarabinose addition could affect the charge interactions between the negatively charged phosphate groups of lipid A and positively charged amino acid residues at the edge of the MD2 hydrophobic pocket. These alterations will reduce binding strength and potentially affect the formation and stability of the lipid A, MD2, and TLR4 signaling complex. We also deleted the late acyl transferases *lpxP* and *msbB* in order to prevent the addition of acyl-oxy-acyl C_16:1_ and acyl-oxy-acyl C_14_, respectively. This alteration could be expected to lock the bacteria into producing tetra-acylated molecules at lower temperatures. Our mass spectra confirmed the lack of aminoarabinose addition in the Δ*phoP* mutant grown at 26°C (strain 44@26) and the lack of C_16:1_ and C_14_ additions in the Δ*lpxP* Δ*msbB* mutant (18, 19) grown at 26°C (strain 48@26) (data not shown). Strain 44@26 predominately produced tetra-acylated lipid A without aminoarabinose additions, in addition to penta-acylated structures (acyl-oxy-acyl C_12_ or C_16:1_) and hexa-acylated structures (acyl-oxy-acyl C_12_ and C_16:1_), as expected. Strain 48@26 predominately produced tetra-acylated lipid A with and without aminoarabinose additions to the phosphate residues, as expected. The addition in *trans* of the lipid A 1-phosphatase gene *lpxE* from *Francisella novicida* (*lpxE*_Fn_) produced the *lpxE*_Fn_-expressing strain 467. The *lpxE* from *F. novicida* was used because it is known to be efficient at removing the 1-phosphate from lipid A and because this BSL2 strain is genetically tractable. When grown at 37°C, this strain predominately produced tetra-acylated, monophosphorylated lipid A with and without aminoarabinose additions, as expected, and its lipid A is expected to show antagonistic properties. The in *trans* expression of the 4′-phosphatase gene *lpxF* from *F. novicida*, along with the repair of the functionally inactive, late acyl-oxy-acyl C_16_ transferase gene *pagP* (repaired *Y. pestis pagP* [*pagP*_Yp_^Rep^]), in *Y. pestis* mutant 470 resulted in the addition of one or two C_16_ acyl chains and the removal of the 4′-phosphate group when grown at 37°C. This resulted in predominately monophosphorylated, penta- and hexa-acylated structures with or without aminoarabinose. *Y. pestis* PagP is more active at 37°C, similar to the activity of PagP in the closely related species *Yersinia pseudotuberculosis* (18), and both penta- and hexa-acylated lipid A structures were found to have monophosphorylated structures.

**TABLE 1:** Bacterial strains and plasmids

Strain/molecule identification no.	Strain or plasmid	Genotype or description	Source or reference
W3110	W3110A	Laboratory *E. coli* K-12 derivative strain, F^−^ λ^−^ *aroA*::Tn*10*	19
358	KIM6+	*Y. pestis* KIM6+, pCD1^−^^a^	18, 19
44	KIM6+ ∆*phoP*	*Y. pestis* KIM6+, pCD1^−^, ∆*phoP*	18
48	KIM6+ Δ*lpxP* Δ*msbB*	*Y. pestis* KIM6+, pCD1^−^, ∆*lpxP* Δ*msbB*	19
467	KIM6+/pWSK29-*lpxE*_Fn_	*Y. pestis* KIM6+, pCD1^−^, carrying pWSK29-*lpxE*_Fn_	This work
	pCVD442-*pagP*_Yp_^Rep^	*sacB* suicide plasmid to facilitate allelic exchange of *pagP*_Yp_^Rep^	This work
470	KIM6+ *pagP*_Yp_^Rep^/pWSK29-*lpxF*_Fn_	*Y. pestis* KIM6+, pCD1^−^, with a repaired *pagP*_Yp_ and transformed by pWSK29-*lpxF*_Fn_	This work
440	KIM6+ Δ*lpxP* Δ*msbB pagP*_Yp_^Rep^	*Y. pestis* KIM6+, pCD1^−^, ∆*lpxP* Δ*msbB pagP*_Yp_^Rep^	This work
439	KIM6+ ∆*lpxP pagP*_Yp_^Rep^	*Y. pestis* KIM6+, pCD1^−^, ∆*lpxP pagP*_Yp_^Rep^	This work

^a^ pCD1^−^, lacking pCD1.

Previous studies have shown that LpxF_Fn_ cannot act upon penta- or hexa-acylated structures with secondary fatty acids at the 3′ position (20). PagP may be adding a C_16:0_ to the 2 position of lipid A for the monophosphorylated, penta-acylated structure and to the 2 and 3′ positions of lipid A after removal of the 4′-phosphate group for the monophosphorylated, hexa-acylated structure (21). We also repaired the C_16:0_ acyl transferase *pagP*_Yp_ in the late acyltransferase Δ*lpxP* and Δ*lpxP* Δ*msbB* mutants (18, 19), which lack the ability to add C_16:1_, or both C_16:1_ and C_12_, respectively, to lipid A to create mutant strains 439 (∆*lpxP pagP*_Yp_^Rep^) and 440 (Δ*lpxP* Δ*msbB pagP*_Yp_^Rep^). When produced by bacteria grown at 37°C, LOS from strain 439 consists mainly of tetra-, C_16:0_ penta-, and (C_16:0_)_2_ hexa-acylated structures. When produced by bacteria grown at 26°C, strain 440 LOS consists mainly of the tetra-acylated structure, with some aminoarabinose additions and C_16:0_ penta-acylated molecules. The presence of the predicted structures was confirmed by mass spectrometry (data not shown).

### Differential activation of cultured mammalian cells by TLR4L structures.

**FIG 2:**
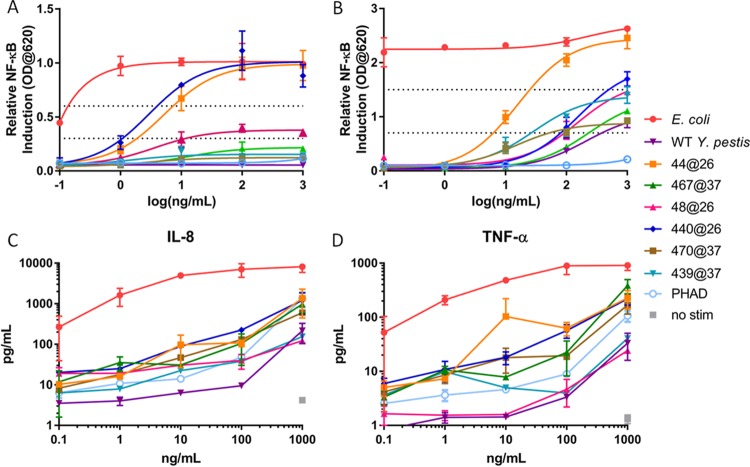
Differential activation of NF-κB by BECC TLR4 ligands. (A and B) HEK-Blue hTLR4 (A) and mTLR4 (B) cells were stimulated with LOS_Ec_, LOSYp@37, LOSYp44@26, LOSYp467@37, LOSYp48@26, LOSYp440@26, LOSYp439@37, or LOSYp470@37 over a 5-log dilution series ranging from 0.1 to 1,000 ng/ml. SEAP secretion was measured in duplicate at an optical density (OD) of 620 nm. Mean results ± SD are graphed with a sigmoidal nonlinear regression line of best fit. (C and D) The same molecules were incubated in a similar manner with THP-1 cells, and cytokine secretion measured using the Millipore Bio-Plex assay. The concentrations of IL-8 (C) and TNF-α (D) are graphed as the mean results ± SD of biological duplicates.

The inflammatory potential associated with LPS is dependent on the structure of the molecule and its affinity for the MD2-TLR4 complex. Specifically, the lipid A structure determines the strength of TLR4 signaling and downstream inflammation by altering both its affinity to MD2, which determines how long the molecule will stay in MD2, and its ability to induce a conformational change in MD2 that favors TLR4 signaling (22). To investigate the capacity of our newly created TLR4Ls to activate NF-κB, several *in vitro* cell culture systems were used. First, HEK-Blue cells that stably coexpress genes encoding either human or murine molecules of the TLR4 signaling complex, TLR4, MD2 and CD14, and an NF-κB-inducible gene encoding a secreted embryonic alkaline phosphatase (SEAP) reporter were used to screen the TLR4Ls. Cells were stimulated over a 5-log concentration range (1 µg/ml to 0.1 ng/ml) for 18 h, and SEAP activity was measured in a colorimetric assay to determine NF-κB activation levels. LOS from *E. coli* (LOS_Ec_) was the positive control in all cell culture experiments. Consistent with previously published observations, the mouse TLR4-MD2-CD14 complex induced a more potent NF-κB response (23). TLR4Ls LOS of *Y. pestis* strain 44@26 (LOSYp44@26) and LOSYp440@26 activated the NF-κB pathway to a level approaching that of LOS_Ec_, whereas LOSYp467@37, LOSYp48@26, LOSYp439@37, and LOSYp470@37 were found to activate both human TLR4 (hTLR4) and mouse TLR4 (mTLR4) cells less than LOS_Ec_ at the maximal dose of 1 µg/ml but still more strongly than wild-type *Y. pestis* LOS grown at 37°C (LOSYp@37) (Table 1; Fig. 2A and B). LOS_Ec_ was a potent NF-κB-activating molecule even at low concentrations, with a 50% effective concentration (EC_50_) of 8.12 × 10^−5^ ng/ml. LOSYp44@26 and LOSYp440@26 had roughly the same levels of maximal activity but were much less potent, with EC_50_ values of 5.235 ng/ml and 2.935 ng/ml, respectively.

To examine the effects of BECC TLR4Ls on human immune cells, we measured the cytokine secretion induced by our candidate molecules from cultured, activated THP-1 human cells. As we cannot take all the candidates through *in vivo* testing, we used *in vitro* screening of induced cytokines to limit the number of candidates. Furthermore, with *in vitro* screening, we can search for profiles similar to that of PHAD, a molecule currently undergoing clinical investigation for use as a vaccine adjuvant, as well as be alerted to activity that may lead to toxicity. Inflammatory pathway activation was investigated by stimulating THP-1 cells for 18 h with the BECC candidates, followed by Bio-Plex analysis of the cytokines IL-8 and tumor necrosis factor alpha (TNF-α). THP-1 cells are a human leukemia monocytic cell line that has been used extensively to estimate the modulation of monocyte and macrophage functional responses (24). To provide context for cytokine secretion data, candidate BECC molecules were compared to PHAD (also known as glucopyranosyl lipid adjuvant [GLA]), a synthetic TLR4 agonist currently being tested clinically in several component vaccines (13–16). PHAD was originally developed for use as an adjuvant based on its ability to drive a Th1 response, which may correlate with protective vaccine efficacy (25). Throughout most of the dose-response curves, LOSYp44@26 and, to a lesser extent, LOSYp440@26 induced cytokine levels comparable to or higher than the levels induced by PHAD (Fig. 2C and D). Due to the generally increased levels of induction of TNF-α and IL-8 compared to the levels induced by PHAD and to favorable NF-κB induction, LOSYp44@26 and LOSYp440@26 were selected for testing in primary cell culture systems.

### Differential activation of primary mammalian cells by TLR4L structures.

**FIG 3:**
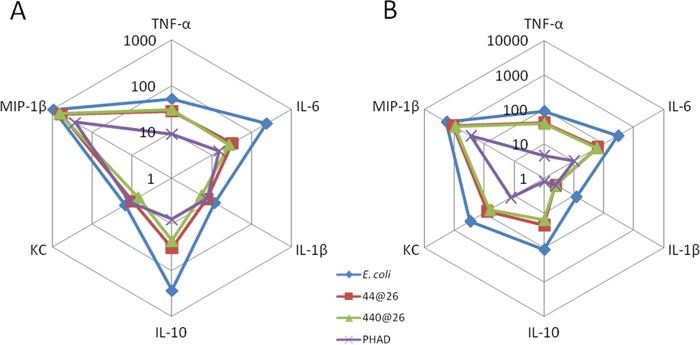
Activation of mouse primary splenocytes by BECC TLR4 ligands. Primary mouse splenocytes pooled from two mice each of either the C57BL6 (A) or BALB/c (B) strain were incubated for 36 h with 100 ng/ml of LOS_Ec_, LOSYp44@26, LOSYp440@26, or PHAD. Cytokine secretion data, as measured by Luminex assay, are shown in pg/ml.

**FIG 4:**
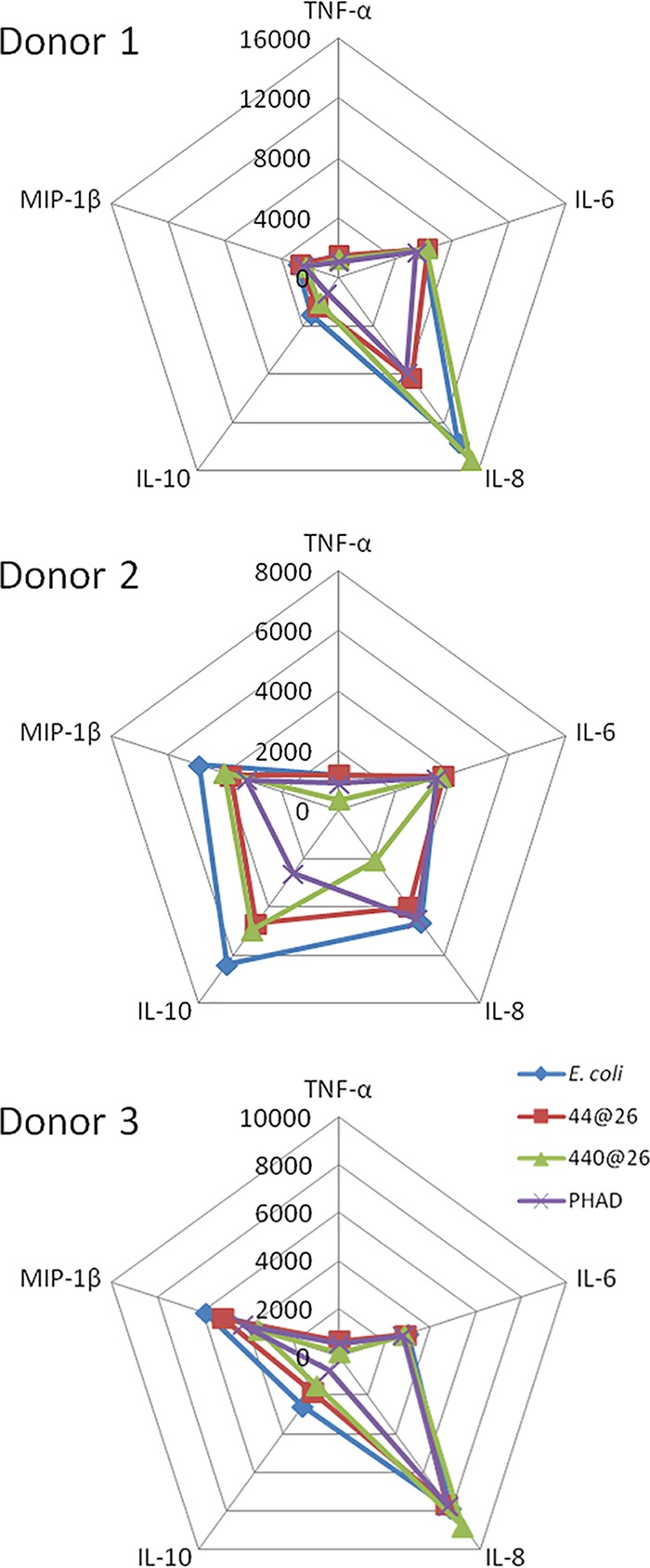
Activation of human primary PBMCs by BECC-derived TLR4 ligands. Primary human PBMCs from three separate donors were incubated for 36 h with 1,000 ng/ml of LOS_Ec_, LOSYp44@26, LOSYp440@26, or PHAD. Cytokine secretion data, as measured by Luminex assay, are shown in pg/ml.

To determine the capability of our novel TLR4Ls to stimulate cytokine responses across genetic backgrounds, primary mouse splenocytes from C57BL/6 and BALB/c strains were isolated and stimulated over a 5-log dose range with LOS_Ec_, LOSYp44@26, LOSYp440@26, or PHAD, and the TNF-α, IL-6, IL-10, KC, IL-1β, and MIP-1β levels were measured. Each analyte measured provides evidence for the stimulation or recruitment of specific immune cells. TNF-α is associated with acute-phase macrophage activation, IL-6 with Th-17 differentiation, IL-10 with T-regulatory cells, KC/IL-8 with neutrophil chemotaxis, and IL-1β with pyrogenic inflammation, and MIP-1β is a potent chemokine for immune cells. Measuring the production of this wide array of molecules allows us to infer the type of immune response that will likely be initiated by BECC TLR4Ls. The trends observed were consistent among the doses tested. A dose-response relationship was observed for each of the analytes tested over a 5-log range. Radar charts plotting cytokine levels from one concentration are shown to allow direct comparison of cytokine levels among each cell population tested (Fig. 3 and 4). At 100 ng/ml in splenocytes isolated from C57BL/6 mice, known to be predisposed to Th1 responses *in vivo*, both LOSYp44@26 and LOSYp440@26 induced a cytokine profile comparable in magnitude to that induced by PHAD but substantially lower than that induced by LOS_Ec_ (Fig. 3A). Furthermore, in splenocytes isolated from BALB/c mice, known to be predisposed for potent Th2-associated immune responses (26), LOSYp44@26 and LOSYp440@26 induced increased levels of cytokines compared to the levels induced by PHAD, but these levels remained lower than the levels induced by LOS_Ec_ (Fig. 3B).

Human peripheral blood mononuclear cells (PBMCs) isolated from venipuncture-collected whole blood from three separate donors were stimulated at 1 μg/ml for 36 h with LOSYp44@26, LOSYp440@26, PHAD, or LOS_Ec_, followed by multiplex analysis of cytokines in the supernatant, including TNF-α, IL-6, IL-8, IL-10, and MIP-1β. All of the TLR4L compounds induced TNF-α and IL-6 production at similar levels (Fig. 4). It is of note that two of the donors exhibited strong proinflammatory IL-8 responses, while the third donor initiated a higher IL-10 response; IL-10 is a cytokine classically associated with immune response dampening. This heterogeneous immune response over even a small cohort of human samples is not surprising, due to genetic and environmental variability (27).

### Lipid A stimulates and activates cytokine secretion similarly to the parent LOS structure.

**FIG 5:**
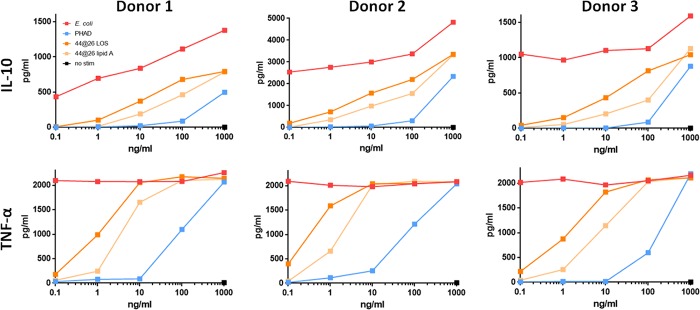
LOS and lipid A initiate similar levels of human PBMC cytokine secretion. Primary hPBMCs from three separate donors were cultured *in vitro* and stimulated for 36 h over a 5-log dose range with LOS_Ec_, LOSYp44@26, LAYp44@26, or PHAD. Data for IL-10 and TNF-α secretion, as measured by Luminex assay, are graphed in pg/ml.

In *Y. pestis*, the LOS is composed of a short carbohydrate (oligosaccharide core) chain bound to lipid A. O antigen is not present in *Y. pestis* due to frameshift mutations in the *ddhB*, *gmd*, *fcl*, and *ushA* genes (28). The lipid A portion of LPS anchors the molecule in the outer membrane of Gram-negative bacteria and is also the portion of the molecule recognized by the TLR4 complex signaling component MD2. To determine whether the core sugars were important in host recognition of the BECC molecules, we removed the core oligosaccharide sugars from LOSYp44@26 by mild acid hydrolysis to yield the lipid A (LAYp44@26) for direct comparison to the full LOS in human primary PBMC stimulation assays (Fig. 5). LAYp44@26 exhibited a slight reduction in the TNF-α and IL-10 responses compared to their induction by the parent LOS molecule. These comparable levels of potency of the lipid A and LOS forms were also observed with several other BECC molecules using our screening methods (data not shown). This observation seems to be different among laboratories and methods used to detect stimulation. However, it is consistent with previous preliminary studies examining the stimulatory properties of lipid A, LOS, and LPS done by our laboratory.

### BECC lipid A upregulates costimulatory markers.

**FIG 6:**
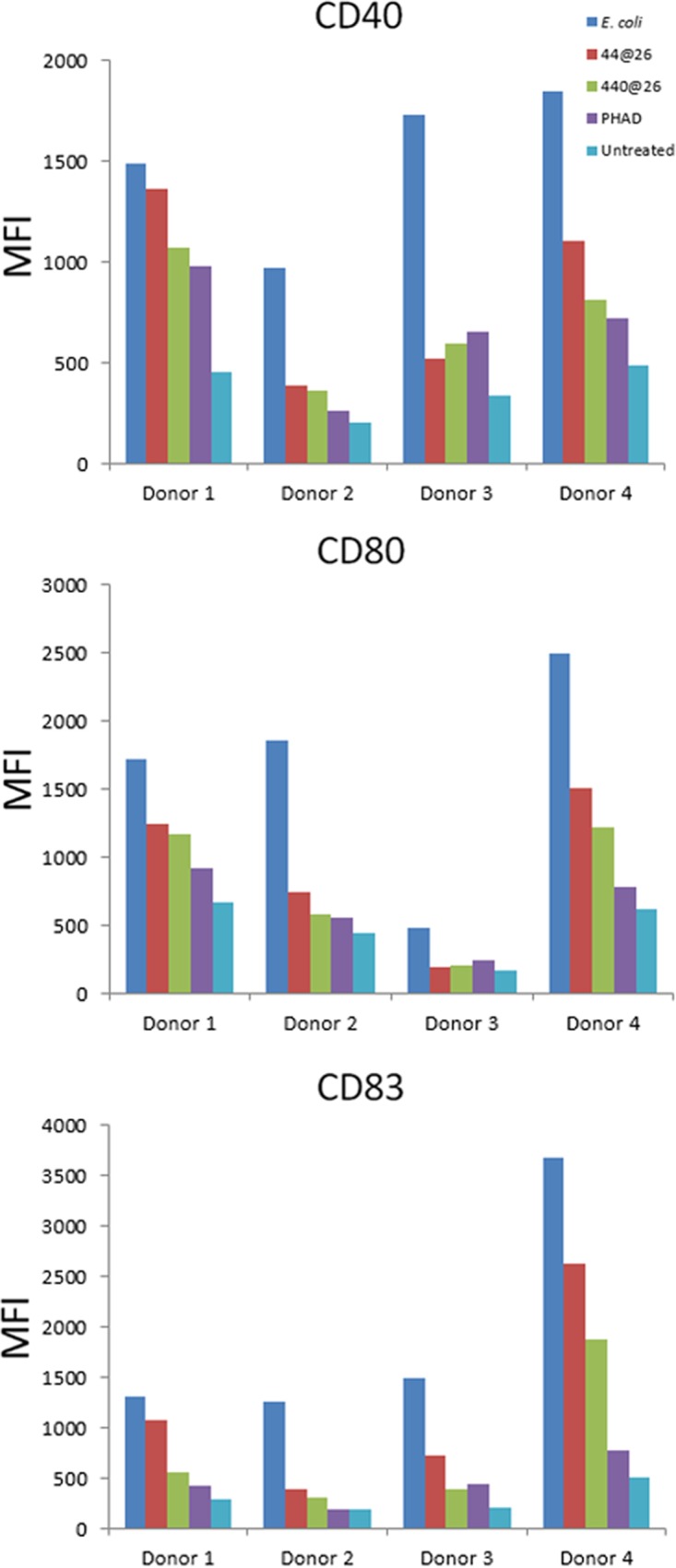
Upregulation of costimulatory surface markers on primary human monocyte-derived dendritic cells upon BECC lipid A stimulation. Primary monocyte-derived dendritic cells from four separate donors were cultured with 1,000 ng/ml of LOS_Ec_, LOSYp44@26, LOSYp440@26, or PHAD. Expression of surface markers CD80, CD83, and CD40 is graphed as mean fluorescence intensity (MFI) as measured by flow cytometry.

Finally, to confirm the potential of TLR4L lipid A structures to initiate an innate immune response, monocyte-derived dendritic cells (DCs) from the four unique human donors were stimulated for 24 h with BECC compounds and analyzed by flow cytometry to determine surface expression of the DC activation markers CD80, CD83, and CD40. TLR4Ls LAYp44@26 and LAYp440@26 were comparable or superior to PHAD in their ability to activate DCs (Fig. 6). As similarly noted in previous human PBMC screenings, variability in the relative expression profiles of specific markers between donors was observed. Specifically, DCs from donors 2 and 3 responded comparably to BECC TLR4Ls and PHAD with regard to immunostimulatory capabilities, while the BECC TLR4Ls were superior to PHAD in the upregulation of costimulatory markers in DCs from donors 1 and 4. The comparable upregulation of costimulatory markers by the BECC TLR4Ls and PHAD, which is an adjuvant molecule under investigation in clinical trials, suggests that these BECC TLR4Ls warrant further investigation into their ability to adjuvant immunogenic proteins using *in vivo* vaccine models.

## DISCUSSION

For effective next-generation vaccines, the identification of high-quality target antigens combined with effective adjuvants will be critical. Adjuvants licensed for use in human vaccines, such as alum and oil-in-water emulsions, are effective but have potential drawbacks. In mice, alum has been shown to stimulate T_H_2-biased IgG1 and IgE antibody production. Alum does not induce strong cell-mediated immunity or high titers of the complement-fixing and virus-neutralizing mouse T_H_1-associated IgG2a and IgG2c subtypes. In both mice and humans, antigen delivered in oil-in-water emulsions can elicit strong and protective T_H_2-based immune responses similarly to alum (29, 30). Depending on which type of pathogen you are vaccinating against, this Th2 response may not always provide the most effective immunity possible. To initiate an immune response more tailored for the desired pathogen, future vaccines will require novel, rationally designed adjuvants that act via defined components of the host innate immune systems, the most promising of which are the Toll-like receptors (TLRs). TLRs are pattern recognition receptors (PRRs) that detect molecules that are broadly shared by pathogens but are structurally distinct from host molecules or are normally compartmentalized away from similar host molecules. Activation of the TLR4 signaling pathway by lipid A can initiate innate immune responses and the development of antigen-specific acquired immunity. The panel of cytokines that are differentially produced can guide the development of the adaptive immune response in different directions: toward Th1 cells, which are effective at combating intracellular infections, T_H_2 cells, which are useful during extracellular parasitic infections, T-regulatory cells (T_reg_s), which are immune response suppressing, or T_H_17 cells, which provide immunity at epithelial/mucosal barriers.

Next-generation TLR4-targeted vaccine adjuvants can be efficiently engineered using the Gram-negative lipid A biosynthesis mechanisms to produce molecules that stimulate specific components of an immune response. Using BECC, we designed and produced a wide variety of lipid A structures, using the attenuated *Y. pestis* strain KIM6+ for expression. These structures were engineered to be structurally intermediate between the highly proinflammatory *E. coli* lipid A and the low-activity, tetra-acylated, wild-type *Y. pestis* lipid A produced at a growth temperature of 37°C (LOSYp@37). This was achieved by adding and/or removing lipid A-modifying enzymes to *Y. pestis* through plasmid transduction. In this study, six BECC-derived molecules were tested for their ability to activate NF-κB (Fig. 2). LOSYp44@26 and LOSYp440@26 were identified as having greater stimulatory properties than LOSYp@37 while retaining dose responsiveness not observed with the highly proinflammatory LOS_Ec_, perhaps due to the presence of tetra-acylated structures known to be antagonistic to the TLR4 signaling pathway (31). We also observed that the cytokine profiles induced by LOSYp44@26 and LOSYp440@26 from the THP-1 monocytic cell line, primary mouse splenocytes from BALB/c and C57BL/6 strains, and human PBMCs were very comparable in magnitude and composition to those induced by PHAD (Fig. 2, 3, and 5). Additionally, the selected BECC compounds were able to induce increased expression of surface costimulatory markers on primary human monocyte-derived DCs (Fig. 6), suggesting that adjuvant properties similar to those of PHAD could be achieved by the use of selected BECC compounds. PHAD was utilized as a comparative control in these assays because of its ability to stimulate the cells in our cell culture systems. MPLA was tested as a possible control for our cell culture models and did not invoke a productive NF-κB response. Due to different mechanisms of action and effective concentrations, alum is also not an informative control for our cell culture assays.

**FIG 7:**
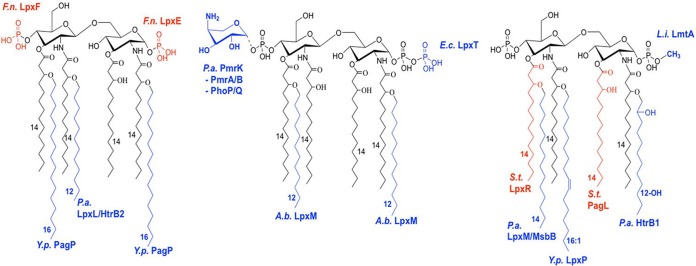
BECC lipid A-modifying enzyme toolbox. Lipid A-modifying enzymes that may be harnessed for modifying *Y. pestis* lipid A are listed next to the associated modification made on *Y. pestis* lipid A grown at 37°C. Red indicates removal, blue indicates addition, and the sources of the enzymes are abbreviated as follows: *F.n.*, *Francisella novicida*; *Y.p.*, *Yersinia pestis*; *P.a.*, *Pseudomonas aeruginosa*; *A.b.*, *Acinetobacter baumannii*; *S.t.*, Salmonella Typhimurium; and *L.i.*, *Leptospira interrogans*.

LOSYp44@26 and LOSYp440@26 have structures with 1 or 2 acyl-oxy-acyl chain additions, conferring a more conical shape to lipid A that could enhance affinity to the MD2 hydrophobic pocket and thus promote a unique response (Fig. 1) (32). LOSYp467@37 and LOSYp48@37 are tetra-acylated like LOSYp@37 and have a cylindrical shape that energetically favors binding to MD2 in a conformation that does not favor TLR4-MD2 complex oligomerization and signaling (32, 33). Conversely, LOSYp439@37 and LOSYp470@37 have hexa-acylated structures with two C_16:0_ acyl-oxy-acyl chain additions, potentially producing a conical, symmetrical lipid A structure that does not efficiently activate TLR4 signaling pathways. The toolbox of enzymes used to create the novel TLR4Ls, shown in Fig. 7, can be expanded as new enzymes are identified that participate in the lipid A synthesis pathways of diverse Gram-negative bacteria. These data warrant further exploration of the relationship between lipid A structure and TLR4 agonist activity for novel lipid A constructs.

This study highlights some of the potential lipid A modifications that can be generated using BECC, including knocking out lipid A-modifying enzymes and key components of regulatory pathways affecting lipid A structure and expressing lipid A-modifying enzymes from heterologous bacteria. *Y. pestis* lipid A was used as the backbone molecule for this study, but other Gram-negative bacteria, such as *Pseudomonas aeruginosa*, have different base lipid A structures, including variations in the length, number, and position of fatty acids, that could affect TLR4 signaling potential. The molecules identified in this screen have strong potential to be successful vaccine adjuvants. Similarly to already-licensed TLR4-based adjuvants, combination with alum during vaccine formulation will likely boost the immunogenic effect. The generation of new and optimized lipid A structures for use as future adjuvants using BECC will continue to be refined as more is known about how the lipid A structure impacts the initial TLR4 signaling pathways, downstream cytokine production, and generation of protective Th1 or Th2 memory responses.

## MATERIALS AND METHODS

### BECC.

To create novel, biologically expressed lipid A structures using bacterial enzymatic combinatorial chemistry (BECC), lipid A-modifying enzyme genes *phoP*, *lpxP*, *msbB*, *lpxE*, *pagP*, and/or *lpxF* were added to or deleted in *Y. pestis* strain KIM6+ in various combinations, described in Table 1. *Y. pestis* KIM6+ expressing *Francisella novicida* LpxE (strain 467) was generated by bacterial conjugation with *E. coli* strain S17-1 λ*pir* containing pWSK29-*lpxE*_Fn_ (34) and was selected using 100 μg/ml carbenicillin. The mutant was confirmed by mass spectrum analysis of lipid A. The Δ*phoP* (strain 44) and Δ*msbB* Δ*lpxP* (strain 48) *Y. pestis* KIM6+ strains were generated previously (18, 19). To generate the strain with repaired *Y. pestis pagP* (*pagP*_Yp_^Rep^), a PCR product containing *Y. pestis pagP* with a base substitution from A to G at position 653 was mutated and amplified by splicing by overhang extension PCR (35). The outer primers were designed to incorporate SacI and SmaI sites. The PCR product and the allelic exchange suicide plasmid pCVD442 (a gift from Michael Donnenberg and James Kaper, University of Maryland—Baltimore; Addgene plasmid no. 11074) (36) were digested with corresponding enzymes, ligated, and transformed into the *E. coli* conjugation donor strain, S17-1 λpir. The pCVD442-*pagP*_Yp_^Rep^ plasmid was confirmed by sequencing and then mated to avirulent *Y. pestis* KIM6+. The merodiploids were selected on *Yersinia* selective agar base (YSAB) (catalog no. CM0653B; Remel, Inc.) supplemented with 100 μg/ml carbenicillin. The merodiploids were struck again for single-colony isolation. Single-colony isolates were grown overnight in brain heart infusion (BHI) broth, followed by plating on Congo red medium supplemented with 7.5% sucrose to select against *sacB* on the integrated plasmid. The sucrose-resistant colonies were then patched onto YSAB with and without carbenicillin, and sucrose-resistant, carbenicillin-sensitive colonies were isolated for sequencing to confirm the presence of the wild-type or repaired *pagP*.

*Y. pestis* KIM6+ *pagP*_Yp_^Rep^ expressing *Francisella novicida* LpxF (strain 470) was generated by bacterial conjugation with *E. coli* S17-1 λ*pir* containing pWSK29-*lpxF*_Fn_ (34) and was selected by using 100 μg/ml carbenicillin. The mutant was confirmed by mass spectrum analysis of lipid A. The previously made Δ*lpxP* and Δ*msbB* Δ*lpxP* strains were transformed with pCVD442-*pagP*_Yp_^Rep^ to generate the Δ*lpxP* Δ*msbB pagP*_Yp_^Rep^ and Δ*lpxP pagP*_Yp_^Rep^ strains (strain 440 and strain 439, respectively). The mutants were confirmed by mass spectrum analysis of lipid A. The associated predicted structures shown in Fig. 1 were drawn using ChemDraw (PerkinElmer).

### Bacterial growth and LOS extraction.

*Y. pestis* strains, stored as glycerol stocks, were grown overnight on BHI agar at 37°C. A single colony was selected for overnight growth with shaking (220 rpm) at 26 or 37°C in 10 ml of BHI supplemented with 1 mM MgCl_2_. Ten milliliters of the overnight culture was then used to inoculate 1 liter of BHI with 1 mM MgCl_2_ prewarmed to either 26 or 37°C and grown to stationary culture under the same conditions for 18 to 24 h. *E. coli* strain W3110 was grown similarly but only at 37°C and in lysogenic broth supplemented with 1 mM MgCl_2_. The bacteria were pelleted, lyophilized, and stored at room temperature until extraction. A proteinase K-digested hot phenol extraction was performed to isolate lipooligosaccharide (LOS) (37). Briefly, 500 mg of lyophilized bacterial pellet was solubilized in a 10 mM Tris-Cl buffer, pH 8.0, with 2% sodium dodecyl sulfate (SDS), 4% β-mercaptoethanol, 20 mg/ml proteinase K, and 2 mM MgCl_2_ at 65°C for 1 h with intermittent vortexing and further digested overnight at 37°C. The samples were precipitated overnight at 20°C with the addition of sodium acetate to a final concentration of 0.1 M and cold ethanol to 75%. LOS was pelleted, and the precipitation was repeated 2 more times to remove residual SDS and peptides. The samples were then suspended in a 10 mM Tris-Cl buffer, pH 7.4, and digested for 4 h at 37°C with 100 μg/ml DNase and 25 μg/ml RNase. An equal volume of 90% phenol was added, and the sample incubated at 65°C for 15 min with occasional vortexing. The sample was cooled in ice-water and centrifuged, and the aqueous fraction was collected. The phenol layer was reextracted with an equal volume of endotoxin-free water. The aqueous layers were pooled and dialyzed in a 1-kDa molecular-mass-cutoff dialysis bag with repeated water changes at 4°C to remove phenol over 48 h. The samples were then frozen on dry ice and lyophilized. Dry samples were washed four times with 2:1 (vol/vol) chloroform-methanol to remove contaminating hydrophilic lipids and reextracted using the Hirschfeld et al. (38) procedure to remove contaminating lipoproteins. Samples were then lyophilized and stored sealed at room temperature.

### Mild acid hydrolysis and mass spectrometry analysis.

LOS was dissolved in 10 mM sodium acetate (Sigma), pH 4.5 (39), to a concentration of 5 to 10 mg/ml and vortexed. This mixture was heated to 100°C for 2 h, cooled on ice, frozen, and lyophilized overnight. The mixture was then washed to remove core sugars and excess salts with the following procedure. Lyophilized samples were suspended in 170 μl of endotoxin-free, cell culture-grade water per 5 to 10 mg of starting LOS, followed by 850 μl of 20 mM HCl in 95% ethanol. Samples were vortexed and centrifuged at 5,000 × *g* for 5 min and then washed in 1 ml of 95% ethanol, followed by centrifugation at 5,000 × *g* for 5 min. These two wash steps were repeated for a total of three times to remove the hydrolyzed core sugars and salt. Samples were suspended in endotoxin-free water and lyophilized in a previously tared tube to determine yield.

Lipid A was solubilized in a 12:6:1 (vol/vol/vol) solution of chloroform-methanol-water and mixed with an equal volume of 20-µg/ml norharmane in 2:1 (vol/vol) chloroform-methanol. One microliter of this mixture was spotted onto a stainless steel target plate. Spectra were gathered in negative mode on a Bruker Microflex matrix-assisted laser desorption ionization–time of flight (MALDI-TOF) mass spectrometry instrument calibrated to an external peptide standard.

### Mouse splenocyte isolation.

Spleens were sterilely harvested from two euthanized naive C57BL/6 and BALB/c mice per strain in a class II biosafety cabinet and placed in sterile 1× phosphate-buffered saline (PBS). Spleens were pushed through a 100-µm cell strainer to create single-cell suspensions, which were then pooled within mouse strains and pelleted in a centrifuge with a swinging bucket rotor. The cell pellet was resuspended in 5 ml of ACK (ammonium-chloride-potassium) lysis buffer and incubated at room temperature for 5 min. Cells were washed and pelleted two more times to ensure removal of red blood cells. Splenocyte pellets were resuspended in Dulbecco's modified Eagle medium (DMEM) and further cultured in stimulation experiments.

### Cell culture and cytokine assays.

All cell culture experiments were incubated at 37°C with 5% CO_2_. HEK-Blue hTLR4 and HEK-Blue mTLR4 cells (Invitrogen) were cultured in DMEM (Gibco) supplemented with 10% heat-inactivated fetal bovine serum (FBS) (Sigma), 100 IU/ml penicillin, 100 µg/ml streptomycin, 1 mM sodium pyruvate, and 200 mM l-glutamine. THP-1 (ATCC), primary mouse splenocytes, and primary human PBMCs (AllCells) were cultured in RPMI 1640 (Gibco) supplemented with 10% heat-inactivated FBS, 100 IU/ml penicillin, 100 µg/ml streptomycin, 1 mM sodium pyruvate, and 200 mM l-glutamine. THP-1 mononuclear cells were cultured with 50 nM vitamin D_3_ (Sigma) for 48 h for activation and differentiation into macrophagelike cells prior to stimulation. PBMCs were isolated from venipuncture-collected whole blood from three separate donors (AllCells). For cell stimulation, lyophilized LOS or lipid A was reconstituted in sterile, endotoxin-free water at a concentration of 1 mg/ml and stored frozen. This stock was serially diluted in the appropriate medium before addition to cell culture. Supernatants were collected from HEK-Blue cells 16 h after stimulation, and the production of SEAP reporter detected using Quanti-Blue (Invitrogen) according to the manufacturer’s instructions. All other cell types were incubated for 36 h before supernatants were collected for measurement of cytokine release. Cytokines were detected from undiluted, cell-free supernatants using custom human and mouse MilliPlex MAP multiplex cytokine assay kits (Millipore) according to the manufacturer’s instructions. Reporter cell line stimulation data were graphed as the mean results ± standard deviations (SD) from biological duplicates and analyzed using GraphPad Prism 7.00 (La Jolla, CA). The log(agonist)-versus-response (three parameters) calculation was run to determine EC_50_s for each of the TLR4 agonists.

### DC maturation and flow cytometry.

The dendritic cell (DC) maturation experiments were contracted to and run by AllCells (Alameda, CA) using standard company protocols. Briefly, human PBMCs from 3 separate donors were cultured in RPMI medium supplemented with 10% FBS, 100 ng/ml human IL-4, and 140 ng/ml human granulocyte-macrophage colony-stimulating factor (GM-CSF) for 7 days, with medium supplementation on day 3. After the 7-day differentiation period, DCs were stimulated with BECC-derived TLR4Ls for 24 h at 37°C, 5% CO_2_. Cells were then lifted off the culture well using Accutase reagent, and EDTA added to halt enzymatic digestion. Cells were washed twice with PBS, nonspecific binding was inhibited using FcBlock (Miltenyi), and finally, cells were stained with CD80-fluorescein isothiocyanate (FITC), CD40-phycoerythrin (PE), and CD83-PE (BD Biosciences). Flow cytometry analysis was performed using an LSRII (BD Biosciences). Dead cells/debris were excluded using a forward scatter/side scatter (FSC/SSC) gate, 10,000 events were collected for each sample, and isotype controls for each antibody were run and determined to contain <1% of events recorded.
